# A heart-coronary arteries structure of carbon nanofibers/graphene/silicon composite anode for high performance lithium ion batteries

**DOI:** 10.1038/s41598-017-09658-4

**Published:** 2017-08-29

**Authors:** Xiaoxin Ma, Guangmei Hou, Qing Ai, Lin Zhang, Pengchao Si, Jinkui Feng, Lijie Ci

**Affiliations:** SDU & Rice Joint Center for Carbon Nanomaterials, Key Laboratory for Liquid-Solid Structural Evolution & Processing of Materials (Ministry of Education), School of Materials Science and Engineering, Shandong University, Jinan, 250061 China

## Abstract

In an animal body, coronary arteries cover around the whole heart and supply the necessary oxygen and nutrition so that the heart muscle can survive as well as can pump blood in and out very efficiently. Inspired by this, we have designed a novel heart-coronary arteries structured electrode by electrospinning carbon nanofibers to cover active anode graphene/silicon particles. Electrospun high conductive nanofibers serve as veins and arteries to enhance the electron transportation and improve the electrochemical properties of the active “heart” particles. This flexible binder free carbon nanofibers/graphene/silicon electrode consists of millions of heart-coronary arteries cells. Besides, in the graphene/silicon “hearts”, graphene network improves the electrical conductivity of silicon nanopaticles, buffers the volume change of silicon, and prevents them from directly contacting with electrolyte. As expected, this novel composite electrode demonstrates excellent lithium storage performance with a 86.5% capacity retention after 200 cycles, along with a high rate performance with a 543 mAh g^−1^ capacity at the rate of 1000 mA g^−1^.

## Introduction

The advanced applications of lithium ion batteries (LIBs) in the fields such as portable electronics, electric vehicles, etc., require higher energy storage capacity with longer cycling life^1^. Silicon has been considered as the most promising materials as anode material of high energy density LIBs to substitute the current commercial graphite anodes, which have been used since the first generation of Sony’s LIBs. It has extremely large theoretical specific energy density of up to 3579 mAh g^−1^ at room temperature, which is 10 times higher than that of graphite materials (372 mA h g^−1^)^2–5^. However, the traditional electrode structure does not fit well for Si materials due to its low electrical conductivity and large volume change (>300%) during lithium ion cycling^6–9^. Cui investigated its related kinetics and fracture behavior by *in-situ* TEM^10^, and found that silicon particles easily fracture if the diameter of Si spheres exceeds a certain size. This causes pulverization of the electrode particles and unceasing formation of solid-electrolyte interphase (SEI) film on their surface^11–13^. As a result, the charging and discharging capacity of this kind of silicon anodes rapidly decline and their cycling life is very poor^14, 15^. On the contrary, nano-sized Si particles only expand and shrink rather than fracture during lithiation and delithiation. Therefore, reducing silicon into various nano-sized structures, such as Si nanoparticles, Si nanowires and Si nanotubes, etc.^16–20^ has been demonstrated as an effective way to alleviate problems induced from the intense volume change.

Interestingly, the expansion and shrinkage of silicon particles is much like a beating heart. Silicon particles expand while lithium ions are transported through the electrolytes to them, which is like the diastole process of a heart while blood flows in it through the coronary arteries. Silicon particles shrink while lithium ions are pulled away from the anodes, which is like the systolic process of a heart while blood is pumped to the whole body. Inspired from this heart-coronary arteries system, we have designed a novel silicon composite structure, in which electrospun carbon nanofibers as the coronary arteries intertwine graphene/silicon (G/Si) particles as the “hearts”. We have demonstrated that the carbon nanofibers intertwined graphene/silicon (G/Si@CFs) structure is a novel binder free anode for LIBs with a much improved long-term cycling stability and a higher specific energy storage capacity.

## Results

### Synthesis and characterization of the heart-coronary arteries structure G/Si@CFs

**Figure 1:**
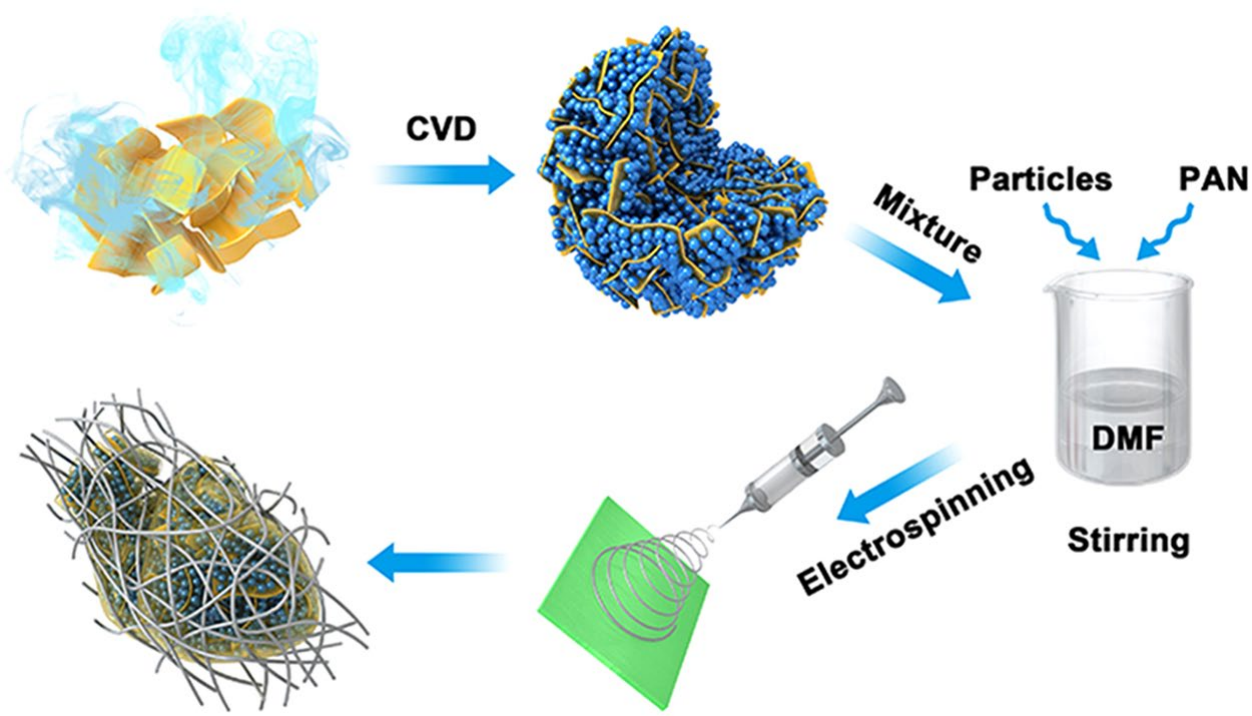
The schematic of the synthesis process of the G/Si@CFs composites. The pictures were drawn by using 3ds Max, Photoshop and ChemDraw.

The preparation procedure for the G/Si@CFs nanocomposite is illustrated in Fig. 1. Graphite oxide (GO) powder was prepared by the Hummer method and a spray drying process^21^. GO powder was then expanded at a high temperature of 1000 °C under N_2_ protection to form micron-sized spheric graphene particles with curved and loose packed graphene sheets. Si nanoparticles were then deposited on the surface of graphene sheets to form G/Si composite particles by a chemical vapor deposition CVD process at 550 °C using silane as Si precursor and hydrogen as carrying gas. Afterward, G/Si particles were mechanically mixed in DMF solution of polyacrylonitrile (PAN), and after the electrospinning process, PAN compounded G/Si particles and PAN nanofibers composite film were prepared. An annealing process at 800 °C in Ar gas was performed, and PAN materials were carbonized to form a composite film of a high conductive carbon nanofibers network and the carbon coated G/Si particles. The PAN based carbon contain heterocyclic nitrogen atoms, which play a critical role in the electronic conductivity between N-doped carbon and adjacent silicon-base materials^22, 23^. The presence of nitrogen atoms can also serve as electron donor to provide electron for better electrochemical properties^24^.

**Figure 2:**
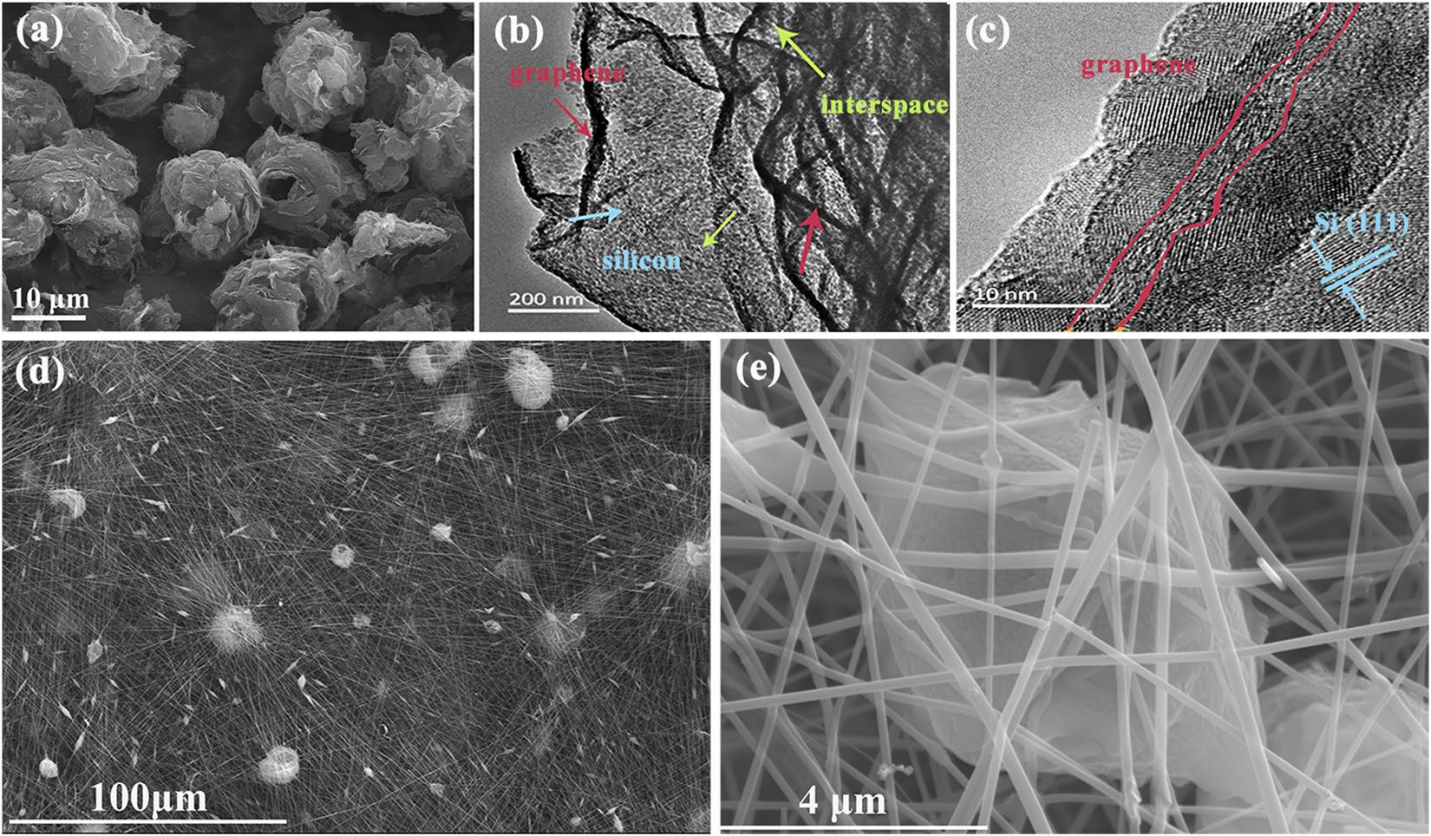
(**a**) SEM image of G/Si particles. (**d**,**e**) Low and high magnitude SEM images of G/Si@CFs. (**b**,**c**) HRTEM image of G/Si partcicles.

**Figure 3:**
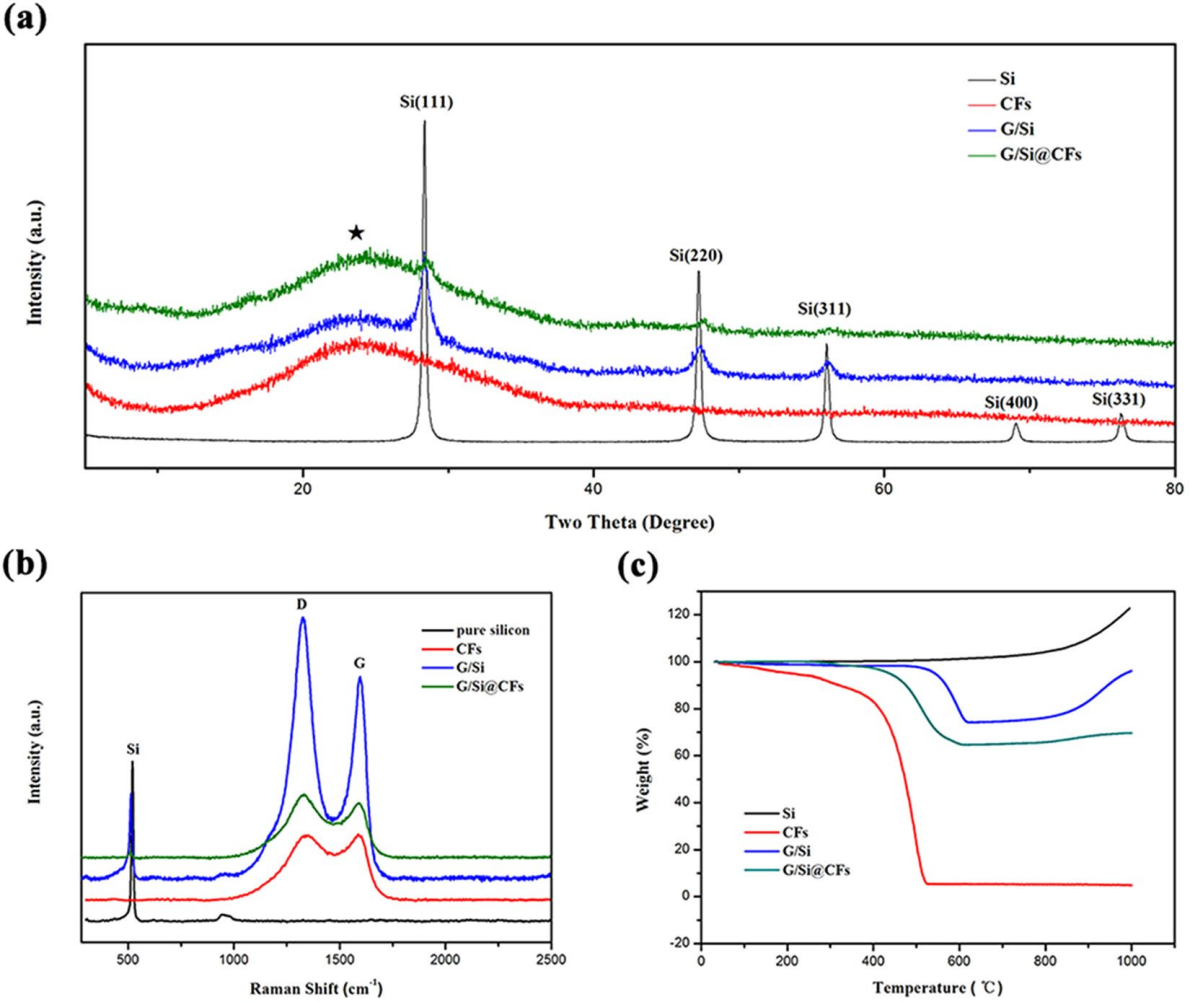
(**a**) XRD patterns. (**b**) Raman spectra and (**c**) TGA curves of CFs, pure silicon nanoparticles, G/Si and G/Si@CFs composites.

Figure 2a shows the scanning electron microscopy (SEM) image of G/Si powders with homogeneous sizes ranging from 5 to 10 μm. From the transmission electron microscopy (TEM) images (Fig. 2b and c), we can see that silicon nanoparticles with an average diameter of 5~10 nm attach on graphene sheets. Those small silicon nanoparticles on electrical conductive graphene sheets constitute the “myocardial cells” of active “hearts” for lithium ions cycling. From the Fig. 2b, the silicon nanoparticles disperse in the middle of the graphene sheets, and the interspaces between the silicon particles can also be clearly observed. In G/Si particles, the graphene sheets link mutually and simultaneously limit the agglomeration of silicon particles. Volume change of silicon during lithium charge and discharge progress will be restrained and buffered by the graphene sheets because nano-sized silicon particles are firmly anchored on their surface (Fig. 2b). Moreover, the high resolution TEM (HRTEM) (Fig. 2c) shows the {111} facets of crystalline silicon with a d-spacing of 0.317 nm, which is consistent with the XRD results of pure silicon (JCPDS card No. 27–1402, Fig. 3a).

Figure 2d and e show the SEM image of G/Si@CFs. The G/Si particles are randomly distributed in the network of carbon nanofibers, and the enlarged image (Fig. 2e) shows that some nanofibers bury in the particle surface. This nanostructure is much like the heart coated by the coronary arteries in the animal body. All the G/Si particles are connected by the carbon nanofibers. Figure 2e show that PAN derived carbon layer on the G/Si particles surface, which is similar to the structure of “pericardium” to avoid directly contacting G/Si particles with electrolyte and help forming a steady SEI film. In this novel structure, surficial carbon coating and flexible conductive graphene frame are both beneficial for the better electrochemical performance of silicon nanoparticles.

The XRD results of pure silicon nanoparticles, G/Si and G/Si@CFs composites are shown in Fig. 3a, the diffraction peaks around 28.5°, 47.4° and 56.3°were indexed to the (111), (220), (311) planes of crystallized Si, respectively. Notably, For the G/Si@CFs, the intensities of Si peaks were weakened, which could be attributed to the coating of carbon. The results also indicate that silicon in the G/Si@CFs remains its crystal structure. There are no observation of impurity peaks like SiC, SiO or SiO_2_. It can be certified that Si didn’t react with carbon or oxygen during the fabrication process. In addition, a broad peak at 2θ = 24.5° in the samples of carbon fibers and G/Si@CFs may represents the amorphous carbon materials derived from PAN, and the intensity of amorphous carbon in G/Si@CFs is equivalent with the intensities of CFs approximately. Raman spectra of different samples are shown in Fig. 3b. The peak at around 520 cm^−1^ is originated from Si, and two distinct peaks observed at 1360 cm^−1^ and 1580 cm^−1^ refer to amorphous (D) and crystallographic (G) modes of carbon, respectively. Moreover, the intensity ratio of D band to G band (I_D_/I_G_) is often used to distinguish the extent of disorder structure of carbon^25^. The I_D_/I_G_ ratios range from 0.99 for CFs to 1.23 for G/Si, while the sample of G/Si@CFs was estimated to be 1.02. This reveals that the pyrolytic PAN derived carbon nanofibers are partially graphitic, which correlates to the enhanced conductivity of the composite electrodes.

### Electrochemical behavior of the G/Si@CFs composite

We also investigated the effects of the G/Si content in the G/Si@CFs composite on the electrochemical performance. The G/Si@CFs anodes composed of 0%, 10%, 20%, 25% and 30% of G/Si were tested at a current density of 0.1 A g^−1^ between 0.01 V and 1.5 V (vs. Li^+^/Li) (Fig. S2) for 100 cycles. In this work, both current densities and specific capacities were calculated on the basis of the total weight of Silicon/carbon composite. From the results we can see that when the content of the G/Si powders in composite anode is below 25%, an excellent long-term cycle stability could be obtained. However, the capacity of the sample with 30% G/Si presents a sudden wastage after 50 cycles, and this may be attributed to the slender nanofibers which can’t bear such a big volume expansion. The carbon nanofibers were broken and lose electrical conductivity. Hence, in order to meet the high capacity and stable long-term cycling, the content of G/Si in G/Si@CFs is designed to be 25 wt% in this paper unless mentioned. In addition, TGA is carried out to calculate the silicon content in the Si/C composite anode in order to accurately evaluate their electrochemical properties, as shown in Fig. 3c. From the thermogravimetric curve of G/Si particles, the silicon content is about 78 wt%. Based on this result, the silicon content of the G/Si@CFs composite anode is calculated to be 20 wt%.

**Figure 4:**
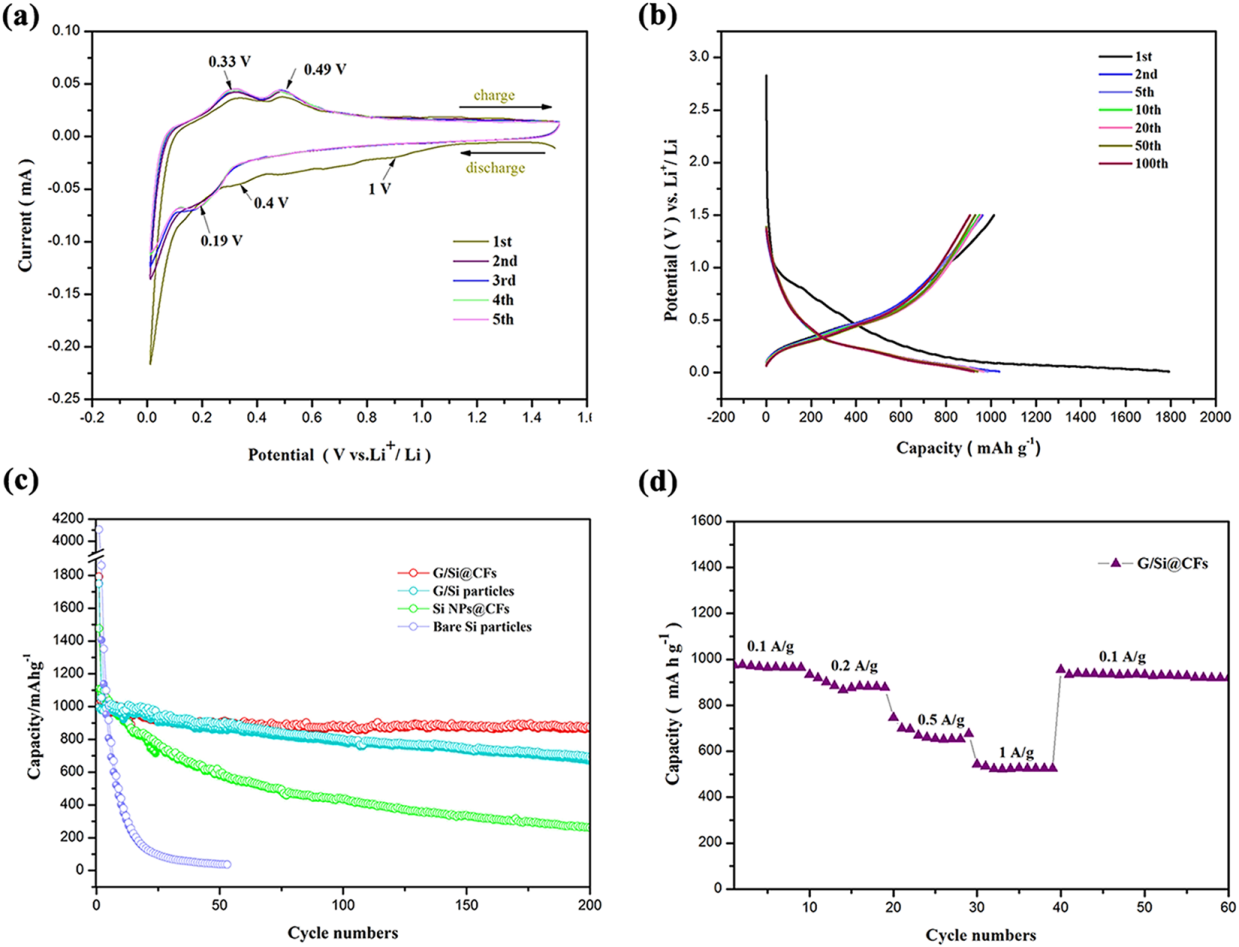
(**a**) CV curves of the first five cycles of the G/Si@CFs electrode. (**b**) Galvanostatic charge-discharge profiles of different cycles for the G/Si@CFs electrode. (**c**) Cycling performance and Coulombic efficiency of the G/Si@CFs, G/Si Particles and Si NP@CFs electrodes. (**d**) Rate capability of the G/Si@CFs and Si NP@CFs electrodes.

The improved electrochemical properties, such as cyclic voltammetry (CV), cycling stability and rate performance of the G/Si@CFs electrodes, were confirmed and shown in Fig. 4. The CV curve can be used to analyze redox behavior during the process of lithiation and delithiation (Fig. 4a). From the CV curve of G/Si@CFs electrode, a small cathodic potential plateau appears at around 0.4 V during the first discharge, corresponding to the irreversible reactions due to the formation of SEI layers. There are a sharp peak below 0.1 V, which is ascribed to the transformation from crystalline Si to Li_x_Si alloy^26, 27^. However, the cathodic peak at 0.4 V disappears in the subsequent cycles, while a new typical discharge peak at about 0.19 V appears indicating alloying between lithium ions and the amorphous silicon. The similar characteristic potential plateaus at 0.33 V and 0.49 V in the anodic sweep for 1st~5th cycles represent the delithiation of the Li_x_Si phase to amorphous Si^28, 29^. More remarkable, with the increasing times of the CV scanning, the current intensity for the cathodic peak and the anodic peak are hardly changed, revealing the lithiation and delithiation of G/Si@CFs is stable during the charge and discharge process. The cycling stability of the G/Si@CFs composite electrode was shown in the Fig. 4b, the composites show an initial discharge capacity of 1792.1 mA h g^−1^ and a charge capacity of 1013.2 mA h g^−1^.

The cycling performance of the heart-coronary arteries structured G/Si@CFs electrode was also compared with that of bare Si particles and G/Si particles at a current density of 100 mA g^−1^ for 100 cycles, as shown in Fig. 4c. Although the first discharge capacity of the bare silicon nanoparticles is high, it declines very fast. The cycling performance of the G/Si@CFs electrode conforms to our assumption for its improved electrochemical performance. The reversible specific capacity (1036.7 mA h g^−1^) of G/Si@CFs electrode is over four times higher than that of commercialized high-performance graphitic anodes. The G/Si@CFs electrode delivered a capacity of 896.8 mA h g^−1^ with a high capacity retention of 86.5% after the 200 cycles, while remaining capacity retention of electrodes of bare silicon nanoparticles and G/Si particles dropped to nearly 0% and 65.5%, respectively. Meanwhile, in order to verify the roles of the “pericardium” on the surface of G/Si “hearts”, we fabricated the silicon nanoparticles/carbon fibers electrodes (Si NP@CFs). As a contrast, the proportion of Si was determined to be 20 wt% in Si NP@CFs composite according the TGA result. The Si NP@CFs presents a poor cycling performance with 23.6% capacity retention after the 100 cycles, as shown in Fig. 4c. The reason may be due to the fact that lots of the Si nanoparticles are exposed on the fiber surfaces and may have severe reaction with electrolyte directly, they drop easily from the nanofiber surface after repeated Li^+^ lithiation and delithiation process. Nevertheless, the carbon shell and the internal graphene sheets of heart-coronary arteries structured G/Si@CFs can prevent Si nanoparticles from exposing in the electrolyte. A higher coulombic efficiency (CE) exceeds 99% of G/Si@CFs electrode was obtained due to the stable SEI film (Fig. S3a). While the CE of G/Si particles fluctuates under 98.5% and the pure pyrolytic CFs keeps a high stable CE at about 99.7%. In addition, the initial coulombic efficiency of the heart-coronary arteries structured anode is higher than of the pure silicon nanoparticles (Fig. S3b). What’s more, comparing the current work with previous works, the heart-coronary arteries structured G/Si@CFs still shows the better cycle performance (see Supporting Information, Table S1).

The rate performance of the G/Si@CFs and Si NP@CFs tested at different current density, increasing from 100 to 1000 mA g^−1^ and subsequently returning to 100 mA g^−1^ (as shown in Fig. 4d). In response to this, the capacity of G/Si@CFs decreases from 974 to 543 mA h g^−1^, then increases back to 957 mA h g^−1^. Therefore, even though the applied current increases tenfold, the discharge capacity of G/Si@CFs still remains at around 55% of its original value. The higher rate performance of G/Si@CFs is attributed to the good electrical conductivity of carbon nanofibers network transferring electron from G/Si “hearts”, just like veins and arteries in human body.

**Figure 5:**
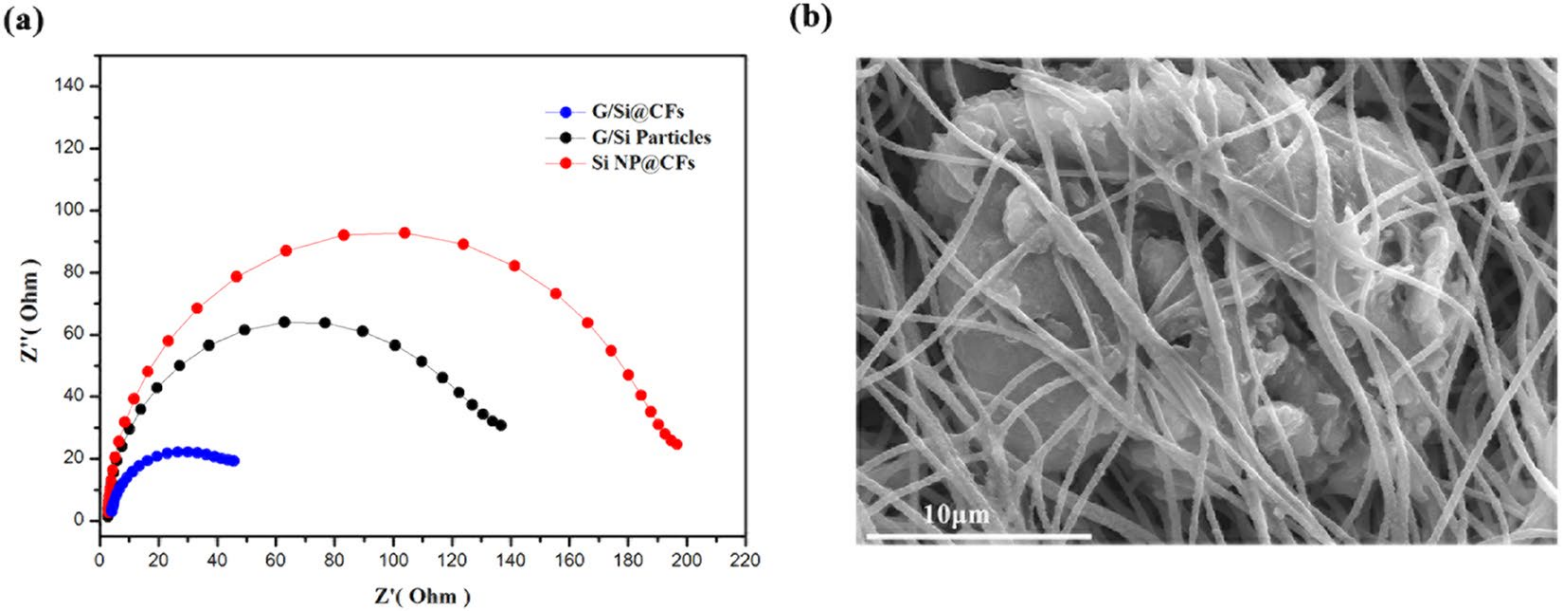
(**a**) Nyquist plots for the semi-circular area in high frequency of the G/Si@CFs, G/Si Particles and Si NP @CFs. (**b**) SEM images of the G/Si@CFs electrode after 100 cycles.

Electrochemical impedance spectroscopy (EIS) was employed to further validate the advantages of the new heart-coronary arteries structured G/Si@CFs. The semicircular area in high frequency of the Nyquist plots is related to charge transfer resistance (R_ct_) of the electrode, electronic resistance as well as solution resistance^30, 31^. As shown in Fig. 5a, the semi-circular diameter of G/Si@CFs is much smaller than those of others, which indicates a much lower electronic resistance within the G/Si@CFs electrode in combination with the ionic resistance of the electrolyte. In addition, SEM imaging of the heart-coronary arteries structured G/Si@CFs electrode after 100 cycles is shown in Fig. 5b. No obvious morphology change were observed, which indicates a remarkable mechanical stability.

## Discussion

As we all known, performance of material is determined by its structure. In this paper, a biomimetic-structured Si/C composite anode inspired from human heart-coronary arteries for lithium ion batteries was designed. Meanwhile, Electrospinning is a new technique to fabricate flexible free-standing electrodes (Fig. S1), which does not need conductive additives, binders and current collector to form a LIB cell^32–35^. This unique structure is able to improve the mass and volume energy density of LIBs and reduce the complexity of manufacturing^36–38^. Si nanoparticles has also been used in the electrospun LIB anode film^39–43^. However, if Si nanoparticles were exposed to the electrolyte, the Si nanoparticles is easy to detach from the electrospun carbon nanofiber surface during cycling, resulting in severe structural damage and capacity loss. Unlike these work, at first time the micrometer-scale graphene/silicon particles combined with the carbon framework were studied, and the newly designed anode realized via electrospinning is free-standing and flexible. Furthermore, in the G/Si particles (Fig. S4), the graphene sheets link mutually and form stable 3D frame structure loaded the silicon nanoparticles. To a certain extents, the padding of silicon particles in the middle of the graphene sheets not only can improve the intensity of the G/Si particles but also sustain the graphene sheets, which is benefit to the stability for the whole structure of the G/Si particles.

In summary, the as-prepared G/Si@CFs composite was used as anodes for LIBs, and outstanding cycling stability and specific energy storage capacity were presented, which is attributed to the special multi-hierarchy carbon framework system, where carbon nanofibers resemble the coronary arteries intertwining G/Si particular “hearts”. With the help of the carbon fibers network, the composite anode electrode is binder-free and flexible, and it has improved cycling life and rate performance with high energy storage capacity. Additionally, the internal graphene framework and the superficial carbon shell of the “heart” structured G/Si particles provide more stable electric pathways and simultaneously prevent the exposure of Si nanoparticles to the electrolyte. The heart-coronary arteries structured G/Si@CFs composites show a discharge capacity of 896.8 mA h g^−1^ after 200 cycles with 86.5% capacity retention at 0.1 A g^−1^, along with a high rate capacity of 543 mA h g^−1^ measured at 1.0 A g^−1^ and remarkable high coulombic efficiency above 99%.

## Method

### Preparation of Graphene/Silicon

Graphene/Silicon (G/Si) composite particles were prepared by a chemical vapor deposition (CVD) method. Graphite oxide (GO) was first prepared by the Hummer method, and after a spray drying process, we got GO powder. GO powder was expanded at a high temperature of 1000 under N_2_ protection to get graphene powder. Si nanoparticles were then deposited on the surface of graphene sheets to form graphene and Si composite particles (G/Si) by a CVD process at 550 °C using Silane as Si precursor and hydrogen as carrying gas.

### Preparation of Graphene/Silicon@Carbon Fibers and Silicon@Carbon Fibers via electrospunn method

1 g of polyacrylonitrile (PAN, (Mw = 150,000 g/mol, Aldrich) and 0.25 g of G/Si particles were mixed in 11.5 g of anhydrous dimethylformamide (DMF, 99.8% purity, Sinopharm Chemical Reagent Co.Ltd). In order to obtain a homogenous precursor solution, the dispersion was violently stirred for 5 h at 50 °C and sonicated for 2 h in a bath-type sonicator. The precursor solution was loaded into a plastic syringe, and electrospinning was conducted by applying a high positive voltage (22 kV). The flow rate of polymer solution is 20 μL min^−1^. The distance from the needle to the copper foil covered plated collector was 15 cm. The as-spun fibers were stabilized at 280 °C for 2 h with a heating rate of 5 °C min^−1^ to remove remaining solvent. The as-spun film was then slowly heated up by 2 °C min^−1^ to 800 °C in Ar flow, and the keeping time is 2 h. For comparative purposes, carbon nanofibers with 20 wt% silicon nanoparticles (<50 nm) were fabricated using the similar method.

### Material characterization

The surface morphology of the as-prepared samples were characterized by field emission scanning electron microscopy (SEM, SU-70) and performed with energy dispersive spectrometer (EDS, HORIBA EX-250). G/Si particles were observed by high resolution transmission electron electroscopy (HRTEM, JEOL JEM-2100). X-ray diffraction (XRD, Rigaku Dmaxrc diffractometer) with CuKa radiation (λ = 0.15418 nm) for 2θ between 10 ° and 80 ° at a scanning rate of 2 ° min^−1^ has been used to characterize the crystal phase of samples. The content of Si in G/Si particles and G/Si@CFs was measured by thermogravimetric analysis (TGA, Mettler-Toledo TGA/SDTA851e) in the temperature range of 30–1000 °C at a heating rate of 5 °C min^−1^ under oxygen. Raman spectra was carried out with monochromatic red light of 633 nm wavelength on room temperature. Carbonized electrospun fibers films were directly treated as negative electrons without additives of any binders and conductors, the G/Si and Si NPs (75 wt%) were mixing with surper P (10 wt%), and CMC (15 wt%) in water to fabricate electrodes for comparison. The slurry was then coated on a Cu foil and dried in a vacuum oven at 100 °C for 12 hours. The obtained anodes were assembled in 2016-type coin cells with electrolyte, which were mixed by LiPF_6_, carbonate (EC) and dimethyl carbonate (DMC) with the volume ratio 1:1:1. In this work, the and the loading mass of the all kind of electrodes is about 0.65–1 mg cm^−2^. The cells measurements were performed galvanostatically within a voltage range of 0.01–1.5 V. Besides, cyclic votammetry (CV, CHI660E) and electrochemical impedance spectroscopy (EIS, Metrohm-Autolab B.V.) were tested at 0.5mVs^−1^ scan rates, and at a frequency range of 100 kHz to 0.01 Hz with a constant perturbation amplitude 5 mV, respectively.

All data generated or analysed during this study are included in this published article (and its Supplementary Information files).

## Electronic supplementary material

Supplementary Materials
