# Supplementary material for: A heart-coronary arteries structure of carbon nanofibers/graphene/silicon composite anode for high performance lithium ion batteries
Source: Sci Rep. 2017 Aug 29;7:9642. doi: 10.1038/s41598-017-09658-4 (PMC5575042; doi:10.1038/s41598-017-09658-4)
Supplement: Supplementary file 1 — Supplementary Materials [file 41598_2017_9658_MOESM1_ESM.pdf]

Supplementary Materials

A heart-coronary arteries structure of carbon nanofibers/ graphene/silicon composite anode for high performance lithium ion batteries

Xiaoxin Ma, Guangmei Hou, Qing Ai, Lin Zhang, Pengchao Si, Jinkui Feng*, Lijie Ci*

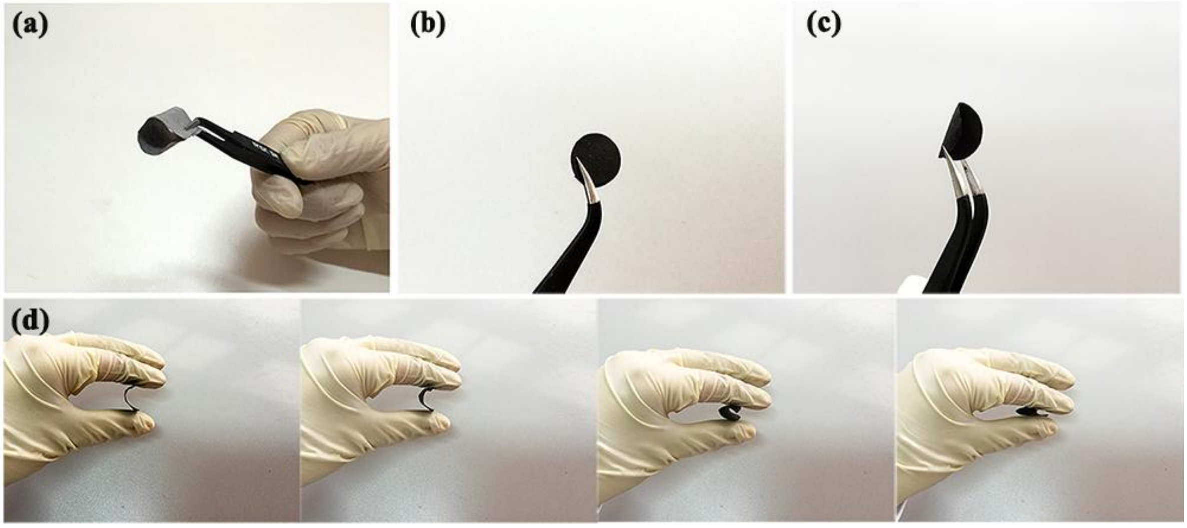

Figure S1. a) as-prepared G/Si@CFs film, b) G/Si@CFs electrode, c) G/Si@CFs electrode after strong bending, d)the carbonized G/Si@CFs film.

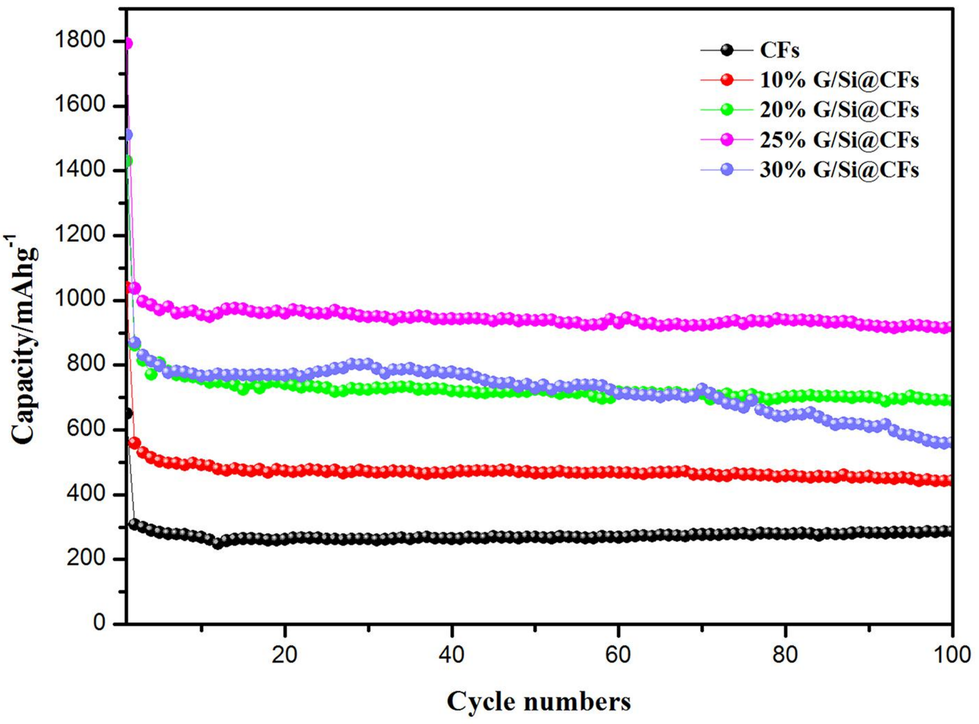

Figure S2. Cycling performance of the G/Si@CFs within different proportion of G/Si particles.

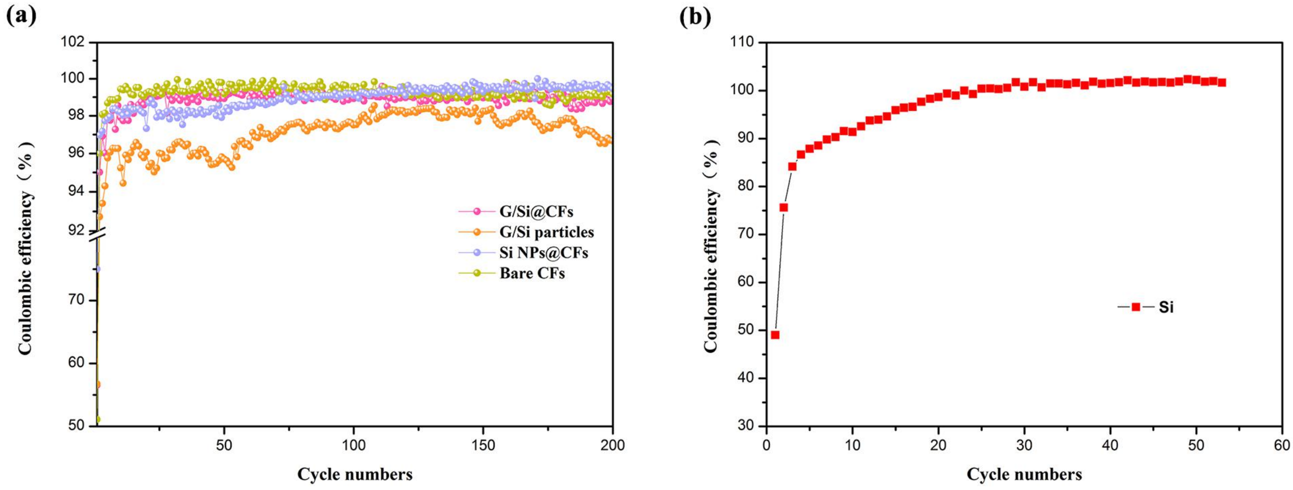

Figure S3. Coulombic efficiency of a) G/Si@CFs, Si NPs@CFs, G/Si, , CFs; b) pure Si particles

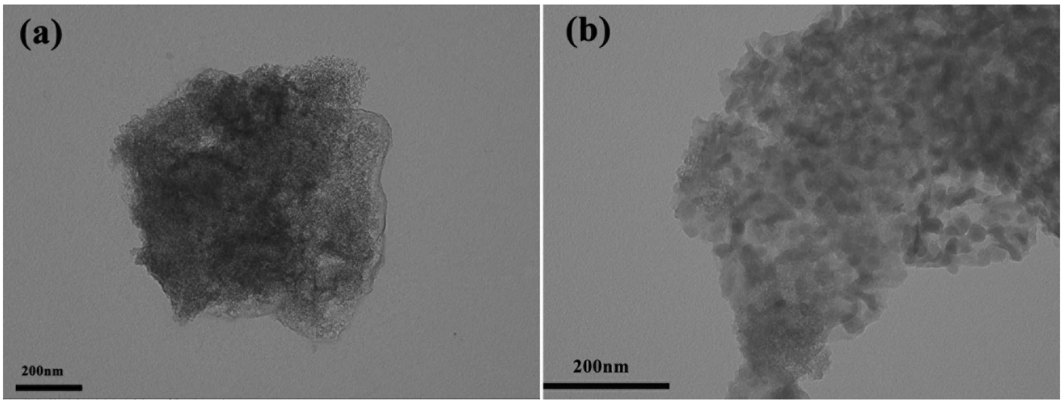

Figure S4 TEM images of the G/Si particles after 100 cycles.

	Current density(mA /g)	Cycle capacity retention /cycling times	cyclic fading rate	
Si/ PCFs	100 mA/g	58%/100cycles	0.42%	Ref 36
G/Si@CFs	100 mA/g	66.3%/50cycles	0.337%	Ref 32
CVD carbon coatings on Si@CNF	50 mA/g	76%/30 cycles	0.8%	Ref 29
TSi/CNF/G	100 mA/g	91%/50 cycles	0.16%	Ref 43
C/Si/AACA	50 mA/g	79%/20 cycles	1.05%	Ref 27
Ground Si@C/CNF	50 mA/g	92%/40 cycles	0.2%	Ref 28
The heart -coronary arteries structuredG/Si@CFs (HCAS G/Si@CFs)	100 mA/g	86.5%/200cycles	0.0675%	Current work

Table S1. Comparison of electrochemical performance between the heart-coronary arteries structured G/Si@CFs and other previous works about Si@Carbon composites as anode materials in LIBs. The current densities and capacities are calculated based on the mass of the composites.
